# Radiographic features of bone ingrowth in highly porous cementless total knee arthroplasty with minimum 2-year follow-up

**DOI:** 10.1186/s42836-026-00422-6

**Published:** 2026-08-10

**Authors:** Kai Chun Augustine Chan, Amy Cheung, Michelle Hilda Luk, Ka Chun Thomas Leung, Chun Man Lawrence Lau, XinYue Chang, Kwong Yuen Chiu, Henry Fu

**Affiliations:** 1 Department of Orthopaedics and Traumatology, School of Clinical Medicine, The University of Hong Kong, Hong Kong, China; 2 Department of Orthopaedics and Traumatology, Queen Mary Hospital, Hong Kong, China; 3 Department of Orthopaedics and Traumatology, Hong Kong Sanatorium Hospital, Hong Kong, China

**Keywords:** Cementless total knee arthroplasty, Total knee arthroplasty, Osteointegration, Biologic fixation, Robotic-assisted TKA, Radiolucent lines, Bone ingrowth, Stress-shielding

## Abstract

**Background:**

The definition of successful osteointegration after cementless total knee arthroplasty (TKA) remains uncertain. We hypothesize that trabecular line formation, bone resorption, and radiolucent line (RLL) disappearance follow a temporal pattern after cementless TKA.

**Methods:**

This is a single-institution retrospective radiographic study involving 221 consecutive patients (301 knees) who had undergone robotic-assisted cementless TKA for primary knee osteoarthritis. All patients had a minimum of 2 years of follow-up (mean 46.5 ± 12.4 months). All TKAs consisted of a 3D-printed titanium-coated tibial tray and cobalt chromium-coated femoral component, which were highly porous, allowing for biologic fixation. Primary outcomes were incidences of trabecular line formation, early radiolucent lines (RLL), and bone resorption. Secondary outcomes were (1) the temporal relationship in RLL disappearance, trabecular line formation, and bone resorption, (2) the relationship between factors with osteointegration, and (3) mid-term clinical outcomes in terms of radiographic alignment, postoperative complications, and revision rates.

**Results:**

Trabecular line formation was observed in 90.4% of knees and was most commonly seen medial (67.2%) and lateral (36.9%) to the tibial keel. RLLs were observed immediately following surgery in 33.6% of knees and were most common at the femoral anterior chamfer region, while bone resorption was most commonly found at the femoral anterior chamfer (41.2%) and the medial tibia (36.5%), indicating a stress shielding effect. Overall, new trabecular line formation occurred early in the postoperative course at an average of 4.5 ± 4.1 months, with bone resorption at a far later stage of 16.2 ± 11.4 months. In knees with early RLL (*n* = 101), disappearance of RLL occurred at a mean of 8.9 ± 8.6 months postop and was preceded by trabecular bridging. In knees without early RLLs (*n* = 200), trabecular line formation occurred at an average of 4.0 ± 3.3 months postop, which occurred at an earlier time point than knees with early RLLs (*p* = 0.009). Timing of bone resorption did not differ significantly between groups (*p* = 0.221). Radiographic alignment was maintained at post-op > 2 years with only two cases (0.7%) of revisions recorded.

**Conclusions:**

In highly porous cementless TKAs with a 3D-printed titanium-coated implant system, bone ingrowth in the form of new trabeculae was observed in > 90% of knees, pointing towards satisfactory osteointegration. Osteointegration typically occurred between 4.5 and 8.9 months, with trabecular line formation and RLL disappearance. Bone remodeling from the stress shielding effect continued beyond 14 months, leading to zones of bone resorption.

**Level of evidence:**

Level III (retrospective study)

## Introduction

End-stage osteoarthritis (OA) often requires surgical intervention in the form of total knee arthroplasty (TKA) when conservative measures fail. Previously, the gold standard for TKA fixation was cemented fixation [1]. However, long-term failure rates in cemented TKAs are concerning, with reported higher risk of aseptic loosening accompanied by failure of the bone-cement interface, which is more evident in young obese patients [2, 3]. With advancements in implant system designs and surgical techniques, there have been reportedly longer survivorships with cementless TKA. Advantages of cementless TKA include shorter operating times, reduced cement-related complications, as well as biologic fixation of implants, leading to increasing interest in cementless TKA globally [4].

Clinical success of cementless TKA largely depends on biologic fixation or osteointegration, where bone growth into the porous prosthetic surface secures the implant [5, 6]. Osteointegration of implants typically takes from 4–12 weeks up to 3 years postoperatively [7]. It involves complex interaction between the prosthetic surface, mesenchymal cells, and migrating osteoblasts [8]. Biologic fixation of cementless components involves both new bone ingrowth and ongrowth. While bone ongrowth is facilitated by grit blasting or plasma spraying of implants, bone ingrowth requires highly porous implants, ideally with pore size between 50–400 μm [9]. Currently, radiostereometric analysis (RSA) provides an extremely precise method for three-dimensional analysis of implant-bone micromotion and assessment of osteointegration [10]. However, complex analysis methods like RSA and artifact-reduction CT require specific radiographic instruments and intraoperative marker bead insertion, which may not be feasible in every centre. Hence, we aim to investigate a simpler and more easily adaptable method for assessment of osteointegration.

Our study aims to investigate the evolution of osteointegration in highly porous cementless TKAs [6] with a minimum of 2 years’ follow-up. Primary outcomes were the incidence of bone ingrowth in the form of trabecular lines, radiolucent lines, and bone resorption following cementless TKA. Secondary outcomes include investigating (1) the temporal relationship of trabecular line formation, disappearance of RLL, and the formation of bone resorption; (2) factors affecting osteointegration; and (3) mid-term clinical outcomes in terms of radiographic alignment, complications, and revision rates.

## Methods

### Study design and setting

This is a single-institution retrospective radiographic study involving consecutive patients who had undergone cementless total knee arthroplasty between January 2019 and September 2023. Patient inclusion criteria were (1) cementless TKA, (2) surgery indicated for primary knee osteoarthritis, (3) robotic-assisted, and (4) usage of the Triathlon Tritanium cementless knee system. Patients were excluded if they had less than 2 years of follow-up or had TKA indicated for secondary osteoarthritis due to trauma, rheumatic arthritis, psoriatic or gouty arthropathy. Radiographs and operation details were retrieved from the electronic medical system. Two investigators (AC and XY) independently graded corresponding radiographs. A consensus meeting was arranged between both investigators before study commencement to standardize radiographic measurements. Neither of the two investigators was involved in any of the operations or postoperative patient care and was blinded to patient information during subsequent measurements. Accuracies of radiographic measurements were further investigated through inter and intra-rater reliability analysis. Before data analysis, any discrepancies in radiographic grading were discussed with a senior orthopaedic surgeon specialized in joint reconstruction (HF) until consensus was reached. Ethical approval from the institutional review board was obtained before study commencement (Ref UW 25-356).

### Participants

A total of 301 knees in 221 consecutive patients (81 men, 140 women) were included, with a mean follow-up of 46.5 ± 12.4 months (range: 24 to 71 months). Patient and surgical details were listed in Table 1.

**Table 1:** Patient demographics and operation details

Patient baseline characteristics	
Gender, N (%)	
Male	81 (36.7)
Female	140 (63.3)
Age at surgery, mean ± SD (range)	65.7 ± 5.9 (48 to 82)
Mean postoperative follow-up, months ± SD (range)	46.5 ± 12.4 (24 to 71)
Body-mass index (BMI), kg/m^2^ ± SD (range)	28.6 ± 4.7 (19.0 to 44.6)
Comorbidities, N (%)	
Hypertension	160 (72.3)
Diabetes mellitus	81 (36.7)
Hyperlipidemia	104 (47.1)
Osteoporosis	5 (2.3)
Malignancy	14 (6.3)
Rheumatic diseases	9 (4.1)
**Operation details**	
Number of knees, N (%)	
One-stage bilateral	80 (36.2)
Unilateral	141 (63.8)
Laterality, N (%)	
Right	157 (52.1)
Left	144 (47.8)
Mean operation time, mins ± SD, range	102.6 ± 29.3 (31 to 224)
Implant type, N (%)	
Cruciate-retaining	281 (93.4)
Posterior stabilising	20 (6.6)

*SD* standard deviation

### Surgical details

All robotic-arm-assisted TKA operations were performed by 6 orthopaedic surgeons of the same academic institution. All operations were done using a standardized technique with the Mako Robotic-Arm-Assisted Surgery System (Mako Surgical Corp, Stryker, USA). All included knees had the Triathlon Tritanium primary cementless total knee implant system (Stryker, USA), which consisted of a 3D-printed titanium-coated tibial tray, a cobalt chromium-coated femoral component, and an X3 polyethylene insert. In-vitro and in vivo studies have verified the biocompatibility of these implants and their capacity to promote osteointegration [11]. Two hundred eighty-one knees received cruciate-retaining, and 20 received posterior-stabilising femoral implants, respectively. All patients had a preoperative CT scan, which allowed for 3D segmentation and surgical planning. No tourniquets were used intraoperatively. All TKAs aimed for a restricted kinematic alignment (rKA) to minimize soft tissue releases. Surgeons prioritized balancing medial and lateral laxity in extension and 90° flexion through fine-tuning individual implant position and aimed for equal gaps medially and laterally as guided by the Mako software. The rKA alignment target adopted was not more than 4° component deviation from neutral and final limb alignment not more than 6° of varus. For knees falling beyond the rKA boundary, soft tissue release in the form of medial collateral ligament (MCL) pie-crusting was performed. All patients underwent a standardized rehabilitation protocol with free weight-bearing walking on day-0 and free mobilization, which included active and passive knee flexion/extension exercises as prescribed by the physiotherapist.

### Postoperative follow-up protocol

Radiographs were routinely taken on postoperative day-1 and subsequently at every follow-up appointment. All radiographs were standardized, and all patients had weight-bearing films taken in both true anteroposterior (AP) and lateral. Standard radiographic protocols and clinical follow-ups were performed at postoperative 2-weeks, 6 weeks, 3-months, 6 months, 12-months, and 2 yearly follow-ups afterwards. In exceptional cases where X-rays were inconclusive or when radiographic quality was suboptimal, radiographs of the nearest follow-up time point were used as a surrogate.

### Variables and outcome measures

**Fig. 1:**
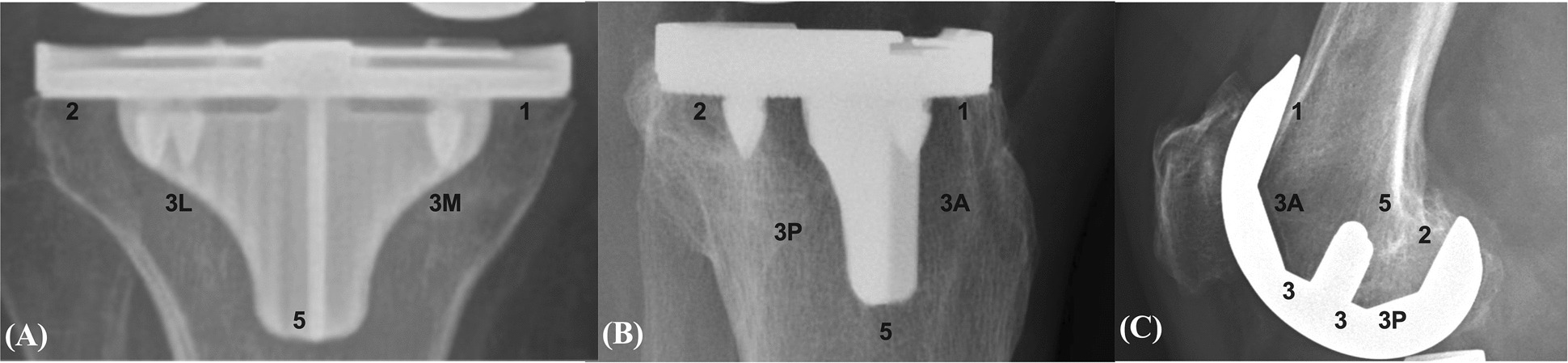
The Modern Knee Society Radiographic Evaluation and Methodology for total knee arthroplasty classification illustrated in anteroposterior (**A**), lateral (**B**) of the tibial component, and lateral view of the femoral component (**C**)

**Fig. 2:**
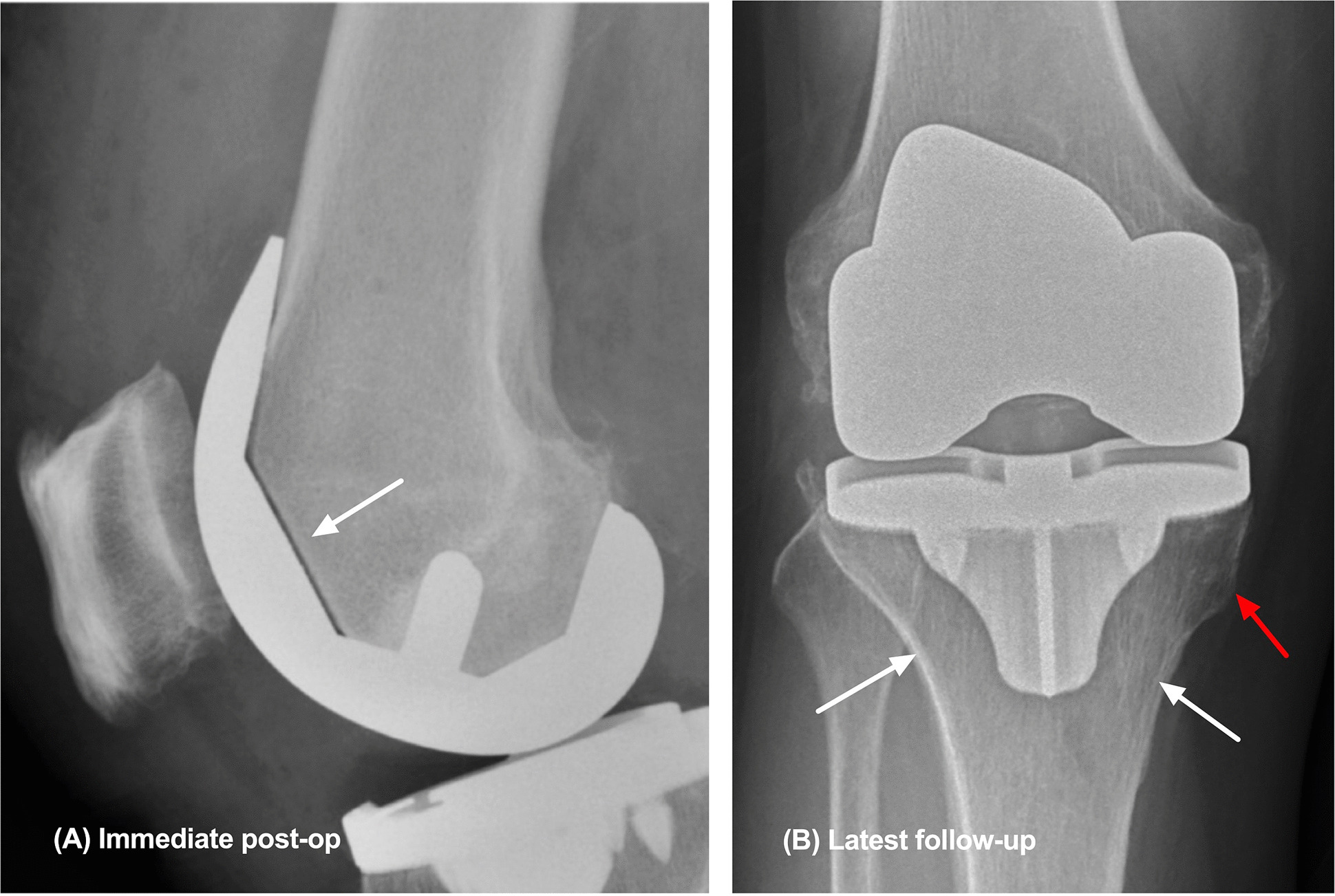
**A** Illustration of early radiolucent line formation at the anterior chamfer region (zone 3 A) of the femoral side in the immediate postoperative period; **B** illustration of new trabecular line formation medial and lateral to the tibial keel (white arrow), and proximal stress shielding leading to bone resorption under the medial tibial tray (red arrow)

Primary outcomes: The incidence of trabecular line formation, early RLLs, and bone resorption were analysed. Trabecular lines are defined as new bone formation absent at immediate postop but gradually develops over time. Radiolucent lines are defined as radiolucent intervals specifically at the bone-implant interface [12]. Early radiolucent lines are specifically identified on the earliest postoperative radiograph [13]. Areas of bone resorption are focal regions of radiolucency that develop at the peri-implant regions. The operational distinction between RLLs and zones of bone resorption was that RLLs appear as a distinct and linear radiolucency while bone resorption usually presents as a diffuse region of reduced bone density. Incidence of RLLs, trabecular lines, and bone resorption was recorded according to the Modern Knee Society Radiographic Evaluation and Methodology for total knee arthroplasty classification [14, 15] (Fig. 1). Both the immediate and latest postoperative periods were compared for formation of trabecular lines, resolution of RLLs, and zones of bone resorption. Examples of these phenomena were depicted in Fig. 2A and B.

Secondary outcomes: The incidence of RLLs and trabecular line formation/bone resorption was recorded at every subsequent follow-up. This longitudinal approach allowed us to understand the temporal relationships between bone ingrowth and disappearance of RLLs, as well as to compare temporal patterns between knees with and without early RLLs.

Exploratory analysis was done to investigate the relationships between factors and trabecular line formation, disappearance of RLLs, and bone resorption. Patient factors included age at surgery, gender, body-mass index(BMI), medical comorbidities(hypertension, hyperlipidemia, diabetes mellitus, rheumatic diseases, osteoporosis, malignancy), smoker/drinker status, intake of calcium supplements, or patient on antiresorptive drugs [16]. Presence of medical comorbidities was retrieved from medical history, while medication history on the hospital electronic medical system was utilised to determine previous antiresorptive use. Osteoporosis was defined as T-score ≤ −2.5 on dual-energy X-ray absorptiometry (DEXA) [17]. Routine DEXA was not performed for elderly patients as part of preoperative workup in our institution.

Mid-term clinical outcomes analysed were changes in individual component alignment in terms of medial proximal tibial angle (MPTA), lateral distal femoral angle (LDFA) and tibial slope. Postoperative complications in terms of aseptic loosening and prosthetic joint infections (PJI) were recorded. Any revision or reoperation procedures were noted.

### Statistical analysis

Continuous variables were compared using the independent-samples t-test, while categorical variables were compared with the *Chi-*square tests. Exploratory analysis on associations between individual factors and osteointegration was conducted with univariate binary logistic regression. Unstandardized beta coefficient (B), odds ratio (OR), 95% confidence intervals (CI), and significance levels were presented. Further multivariate binary logistic regression incorporating all relevant factors was performed to verify significant associations from univariate analysis.

Inter-rater and intra-rater reliabilities were assessed by Cohen’s kappa (κ) for categorical variables (presence/absence of RLLs/trabecular lines/bone resorption). Inter-rater reliability for continuous variables (timing of osteointegration events) was evaluated with the intraclass correlation coefficient (ICC). Inter-rater reliability was assessed by randomly selecting 30 patient radiographs and comparing radiographic measurements by the two investigators (AC and XY), while intra-rater reliability was assessed by repeated same X-ray measurements two weeks apart. The random selection of 5–10% of measurements from the study cohort has been commonly utilised in previous studies for reliability assessment [18, 19]. Fair, moderate, substantial and perfect reliability were defined as having a κ value between 0.21 and 0.40, between 0.41 and 0.60, between 0.61 and 0.80 and between 0.81 and 1.00, respectively [20]. On the other hand, ICC values of 0.5 to 0.75, between 0.75 and 0.90, and above 0.90 were defined as moderate, good, and excellent reliability [21]. Perfect agreement was demonstrated for identification of postoperative RLLs (κ = 1.000), while fair-to-substantial inter-rater reliability was demonstrated for trabecular lines (κ = 0.326 to 0.703) across different zones. Substantial-to-perfect inter-rater reliability was also demonstrated for different zones of bone resorption (κ = 0.875 to 1.000). Substantial-to-perfect intra-rater reliability for RLLs (κ = 1.000), trabecular lines (κ = 0.600 to 1.000), and zones of bone resorption (κ = 0.824 to 1.000) was also demonstrated. The ICC for timing of RLL disappearance, trabecular line formation, and bone resorption were 0.929, 0.960, and 0.981, respectively. Two-tailed significance was set at *p* < 0.05. All statistics were conducted with SPSS Statistics (ver 29.0; IBM, USA).

## Results

### Incidences of trabecular lines, radiolucent lines and bone resorption

A total of 272 knees (90.4%) had varying degrees of new trabecular line formation. They were most frequently found medial (tibial anteroposterior (AP) zone 3 M; 67.2%) and lateral to the tibial keel (tibial AP zone 3L; 36.9%), at the posterior tibial keel (lateral tibial zone 3P; 36.2%) and under the posterior tibial tray (lateral tibial zone 2; 23.6%). Only 17.9% of knees had new trabecular line formation at the femoral side (Table 2). Early RLLs manifested in 33.6% (101/301) of knees. RLLs were most common at the anterior chamfer region (Femoral zone 3 A; 13.4%). For the tibial side, RLLs were more common under the posterior tibial tray (Lateral tibial zone 2; 5.6%) (Table 2). At latest follow-up, 96.0% (97/101) of RLLs spontaneously resolved, and only 4.0% (4/101) persisted. Focal radiolucencies indicating bone resorption occurred in 47.5% of knees (143/301) and were most common at the femoral anterior chamfer region (41.2%). At the tibial side, the medial (36.5%) and anterior tibial tray (36.9%) had more bone resorption (Table 2).

**Table 2:** Incidences of early radiolucent lines at immediate post-op, trabecular line formation and bone resorption at latest follow-up

Implant zones	Trabecular lines, N (%)		Early radiolucent lines, N (%)		Bone resorption, N (%)	
	**Latest follow-up** **(*n* = 301)**	*p*-value^*^	**Immediate post-op** **(*n* = 301)**	*p*-value^*^	**Latest follow-up** **(*n* = 301)**	*p*-value^*^
**Tibial (AP)**	242 (80.4)		16 (5.3)		114 (37.9)	
1	65 (21.6)	< 0.001^#^	11 (3.7)	< 0.001^#^	110 (36.5)	< 0.001^#^
3M	202 (67.2)		1 (0.3)		0	
5	24 (8.0)		0		1 (0.3)	
3L	111 (36.9)		0		1 (0.3)	
2	18(6.0)		7 (2.3)		17 (5.6)	
**Tibial (lat)**	179(59.5)		31 (10.3)		114 (37.9)	
1	34 (11.3)	< 0.001^#^	12 (4.0)	< 0.001^#^	111 (36.9)	< 0.001^#^
3A	49 (16.3)		1 (0.3)		16 (5.3)	
5	5 (1.7)		0		0	
3P	109 (36.2)		0		5 (1.7)	
2	71 (23.6)		17 (5.6)		9 (2.9)	
**Femoral**	54 (17.9)		76 (25.2)		139 (46.2)	
1	20 (6.6)	< 0.001^#^	10 (3.3)	< 0.001^#^	53 (17.6)	< 0.001^#^
3A	34 (11.3)		43 (14.3)		124 (41.2)	
3	3 (1.0)		17 (5.6)		0	
5	6 (2.0)		0		0	
3P	8 (2.7)		9 (3.0)		8 (2.7)	
2	5 (1.7)		4 (1.3)		2 (0.7)	

^*^p-value calculated from *Chi*-square test

^#^statistical significance

### Temporal pattern of osteointegration events

**Fig. 3:**
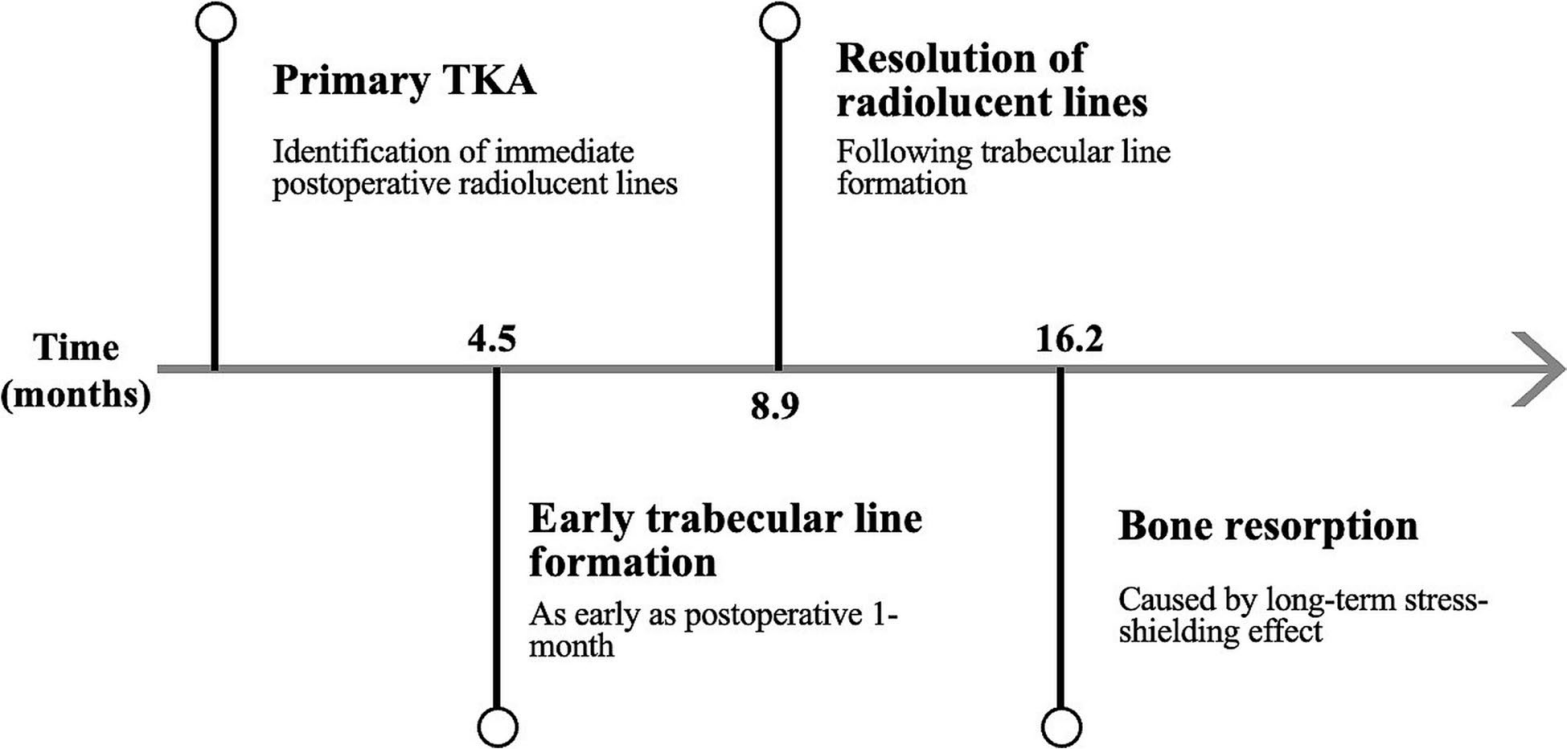
Timeline illustrating temporal relationship of osteointegration events

**Fig. 4:**
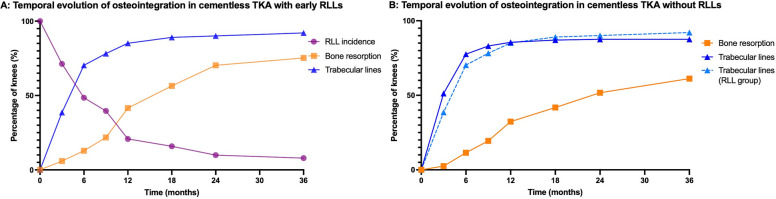
Graphs showing **A** Temporal evolution of events of osteointegration in patients with early radiolucent line (RLL) formation; **B** Temporal evolution of events of osteointegration in patients without early RLLs

Overall (*n* = 301), new trabecular line formation occurred early in postoperative course at an average of 4.5 ± 4.1 months, with bone resorption at a far later stage of 16.2 ± 11.4 months (Fig. 3). In knees with early RLL (*n* = 101), trabecular line formation occurred at an average of 5.5 ± 5.1 months postop, which preceded disappearance of RLL which was at a mean of 8.9 ± 8.6 months postop (*p* < 0.001). Bone resorption often occurred at a later stage, at an average of 14.9 ± 10.5 months postop14.9 ± 10.5 (*p* < 0.001 compared to previous time-points) (Fig. 4A and B). In the RLL subgroup, early trabecular line formation ≤ 5.5 months, as defined by our study results above, predicted early RLL disappearance ≤ 8.9 months (Beta coefficient(B) = 1.368; OR 3.93; 95% CI 1.58–9.75; *p* = 0.003) with binary logistic regression analysis. In knees without early RLLs (*n* = 200), trabecular line formation occurred at an average of 4.0 ± 3.3 months postop while bone resorption occurred at 16.9 ± 11.9 months. Earlier trabecular line formation was observed in knees without early RLLs compared to those with RLLs (*p* = 0.009), while timing of bone resorption did not differ significantly (*p* = 0.221) (Fig. 4A and B).

### Relationships of factors with osteointegration

With univariate analysis, higher BMI (OR 0.943; *p* = 0.016) and hypertension (OR 0.447; *p* = 0.006) were associated with less bone resorption at final follow-up. Relationships between osteoporosis/antiresorptives use and osteointegration events were not significant. The presence of smoking status (12.6% of patients), chronic drinking (14.3%), gender, and age was not associated with osteointegration (Table 3). After multivariate adjustment, only age showed predictive value towards trabecular formation (OR 1.106; *p* = 0.041). Relationship between bone resorption and hypertension was not significant after adjustment (OR 0.439; *p* = 0.076) (Table 4).

**Table 3:** Univariate binary logistic regression analysis investigating relationship between factors and osteointegration

**Factors**		**B**	**S.E**	**Wald**	**OR, 95% CI**	***p*-value**
**Trabecular lines formation (*n* = 301)**						
**Gender (male)**		− 0.395	0.392	1.016	0.674 (0.312 to 1.452)	0.313
**Age at surgery**		0.044	0.038	1.337	1.045 (0.970 to 1.126)	0.248
**BMI**		0.044	0.032	1.922	1.045 (0.982 to 1.112)	0.166
**Smoking status (12.6%)**		1.307	1.038	1.584	3.695 (0.483 to 28.3)	0.208
**Chronic drinker (14.3%)**		− 0.402	0.533	0.571	0.669 (0.235 to 1.900)	0.450
**Comorbidities**						
	Hypertension	0.186	0.424	0.192	1.204 (0.525 to 2.763)	0.661
	Diabetes mellitus	− 0.082	0.403	0.041	0.922 (0.418 to 2030)	0.840
	Hyperlipidemia	0.260	0.396	0.431	1.297 (0.597 to 2.819)	0.512
	Osteoporosis	− 1.189	0.841	1.998	0.305 (0.059 to 1.583)	0.157
	Background malignancy	19.037	> 100	0.000	> 100	0.998
	Rheumatic disease (e.g., rheumatic arthritis)	0.166	1.063	0.024	1.180 (0.147 to 9.483)	0.876
**Others**						
	On antiresorptives	− 0.872	1.136	0.590	0.418 (0.045 to 3.869)	0.442
	On calcium/vitamin D supplements	0.438	0.760	0.332	1.549 (0.350 to 6.684)	0.564
**Disappearance of radiolucent lines (*n* = 101)**						
**Gender (male)**		0.568	1.242	0.209	1.765 (0.155 to 20.119)	0.647
**Age at surgery**		− 0.245	0.154	2.554	0.782 (0.579 to 1.057)	0.110
**BMI**		0.286	0.185	2.388	1.331 (0.926 to 1.913)	0.122
**Smoking status (12.6%)**^**#**^		18.053	12,118	0.000	> 100	0.999
**Chronic drinker (14.3%)**^**#**^		18.082	> 100	0.000	> 100	0.999
**Comorbidities**						
	Hypertension	− 18.011	> 100	0.000	0.000	0.998
	Diabetes mellitus	− 1.386	1.244	1.242	0.250 (0.022 to 2.862)	0.265
	Hyperlipidemia	0.818	1.242	0.434	2.267 (0.199 to 25.842)	0.510
	Osteoporosis ^#^	17.748	> 100	0.000	> 100	1.000
	Background malignancy ^#^	17.8	> 100	0.000	> 100	0.999
	Rheumatic disease (e.g., rheumatic arthritis) ^#^	17.8	> 100	0.000	> 100	0.999
**Others**						
	On antiresorptives^#^	17.8	> 100	0.000	> 100	1.000
	On calcium/vitamin D supplements^#^	17.8	> 100	0.000	> 100	1.000
**Bone resorption (*n* = 301)**						
**Gender (male)**		0.173	0.249	0.514	1.195 (0.734 to 1.945)	0.473
**Age at surgery**		0.028	0.022	1.639	1.028 (0.985 to 1.074)	0.200
**BMI**		− 0.059	0.024	5.808	0.943 (0.899 to 0.989)	0.016*
**Smoking status (12.6%)**		0.397	0.453	0.769	1.488 (0.612 to 3.616)	0.381
**Chronic drinker (14.3%)**		− 0.022	0.389	0.003	0.979 (0.457 to 2.097)	0.956
**Comorbidities**						
	Hypertension	− 0.805	0.296	7.419	0.447 (0.250 to 0.798)	0.006*
	Diabetes mellitus	0.024	0.251	0.009	1.025 (0.626 to 1.677)	0.923
	Hyperlipidemia	− 0.350	0.242	2.092	0.704 (0.438 to 1.132)	0.148
	Osteoporosis	− 0.101	0.740	0.019	0.904 (0.212 to 3.857)	0.891
	Background malignancy	− 0.312	0.481	0.419	0.732 (0.285 to 1.881)	0.517
	Rheumatic disease (e.g., rheumatic arthritis)	− 0.285	0.598	0.227	0.752 (0.233 to 2.430)	0.634
**Others**						
	On antiresorptives	− 2.029	1.125	3.255	0.131 (0.015 to 1.191)	0.071
	On calcium/vitamin D supplements	− 0.070	0.400	0.031	0.932 (0.426 to 2.041)	0.861

^*^Statistical significance

*B* beta coefficient, *S.E* standard error, *Wald* Wald statistic, *OR* odds ratio, *BMI* body-mass index

^#^Logistic regression estimates may be unstable for rare events or sparse categories, and should be interpreted with caution and treated as exploratory

**Table 4:** Multivariate binary logistic regression analysis investigating relationship between factors and osteointegration

**Factors**		**B**	**S.E**	**Wald**	**OR, 95% CI**	***p*-value**
**Trabecular lines formation (*n* = 301)**						
**Gender (male)**		0.168	0.717	0.055	1.183 (0.290 to 4.823)	0.815
**Age at surgery**		0.101	0.049	4.163	1.106 (1.004 to 1.219)	0.041*
**BMI**		0.078	0.043	3.275	1.081 (0.994 to 1.177)	0.070
**Smoking status (12.6%)**		1.158	1.164	0.990	3.184 (0.325 to 31.155)	0.320
**Chronic drinker (14.3%)**		− 0.874	0.806	1.175	0.417 (0.086 to 2.026)	0.417
**Comorbidities**						
	Hypertension	− 0.594	0.711	0.699	0.552 (0.137 to 2.223)	0.403
	Diabetes mellitus	− 0.379	0.588	0.416	0.685 (0.216 to 2.166)	0.519
	Hyperlipidemia	0.294	0.587	0.251	1.342 (0.425 to 4.235)	0.616
	Osteoporosis	− 1.325	1.600	0.685	0.266 (0.012 to 6.121)	0.408
	Background malignancy ^#^	18.855	> 100	0.000	> 100	0.999
	Rheumatic disease (e.g., rheumatic arthritis) ^#^	18.259	> 100	0.000	> 100	0.999
**Others**						
	On antiresorptives^#^	1.264	> 100	0.165	3.540 (0 to > 100)	1.000
	On calcium/vitamin D supplements	0.449	1.104	0.165	1.566 (0.180 to 13.640)	0.685
**Disappearance of radiolucent lines (*n* = 101)**						
**Gender (male)**^**#**^		− 4.179	7.705	0.294	0.015 (0 to > 100)	0.588
**Age at surgery**		− 0.220	0.261	0.709	0.803 (0.481 to 1.339)	0.400
**BMI**		0.370	0.298	1.536	1.447 (0.807 to 2.596)	0.215
**Smoking status (12.6%)**^**#**^		22.122	> 100	0.000	> 100	0.998
**Chronic drinker (14.3%)**^**#**^		16.286	> 100	0.000	> 100	0.998
**Comorbidities**						
	Hypertension^#^	− 21.403	> 100	0.000	0.000	0.998
	Diabetes mellitus	− 3.051	2.174	1.971	0.047 (0.001 to 3.349)	0.160
	Hyperlipidemia	5.427	7.901	0.472	0.047 (0.001 to 3.349)	0.492
	Osteoporosis ^#^	19.696	> 100	0.000	> 100	1.000
	Background malignancy ^#^	9.922	> 100	0.000	> 100	1.000
	Rheumatic disease (e.g., rheumatic arthritis) ^#^	9.526	> 100	0.000	> 100	1.000
**Others**						
	On antiresorptives	− 1.792	> 100	0.000	0.167	1.000
	On calcium/vitamin D supplements^#^	18.536	> 100	0.000	> 100	0.999
**Bone resorption (*n* = 301)**						
**Gender (male)**		− 0.006	0.429	0.000	0.994 (0.429 to 2.303)	0.988
**Age at surgery**		− 0.014	0.031	0.202	0.986 (0.927 to 1.048)	0.653
**BMI**		− 0.037	0.033	1.259	0.963 (0.902 to 1.028)	0.262
**Smoking status (12.6%)**		0.400	0.555	0.521	1.492 (0.503 to 4.425)	0.470
**Chronic drinker (14.3%)**		− 0.255	0.506	0.254	0.775 (0.287 to 2.090)	0.614
**Comorbidities**						
	Hypertension	− 0.822	0.463	3.152	0.439 (0.177 to 1.089)	0.076
	Diabetes mellitus	− 0.123	0.350	0.123	0.885 (0.446 to 1.756)	0.726
	Hyperlipidemia	− 0.182	0.350	0.123	0.833 (0.420 to 1.655)	0.603
	Osteoporosis ^#^	19.9	> 100	0.000	> 100	0.999
	Background malignancy	− 0.367	0.700	0.275	0.693 (0.176 to 2.734)	0.600
	Rheumatic disease (e.g., rheumatic arthritis)	0.942	1.126	0.263	2.565 (0.282 to 23.3)	0.403
**Others**						
	On antiresorptives^#^	− 42.604	> 100	0.000	0.000	0.999
	On calcium/vitamin D supplements	0.288	0.561	0.263	1.333 (0.444 to 4.005)	0.608

^*^Statistical significance

*B* beta coefficient, *S.E* standard error, *Wald* Wald statistic, *OR* odds ratio, *BMI* body-mass index

^#^Logistic regression estimates may be unstable for rare events or sparse categories, and should be interpreted with caution and treated as exploratory

### Mid-term clinical outcomes

There were no statistically significant differences comparing immediate and latest component alignment in terms of MPTA (90.2 ± 1.9 VS 89.9 ± 2.0; *p* = 0.110), LDFA (85.6 ± 2.4 VS 86.0 ± 2.3; *p* = 0.077) and tibial slope (2.3 ± 1.6 VS 2.3 ± 1.7; *p* = 0.790). Two knees (0.7%) had aseptic loosening, one involving the femoral component and the other involving the tibial tray. One knee with femoral side loosening was revised with isolated femoral revision, while another knee was revised with isolated polyethylene exchange for chronic instability without signs of radiological loosening. For the knee revised for chronic instability, physical examination showed mild posterior sag with a grade 2 posterior drawer test as well as grade 2 lateral laxity and a recurvatum of 10°. There were no signs of periprosthetic infection, as minimal clear joint fluid was yielded. Intraoperatively, the tibia was subluxed anteriorly for removal of the previous cruciate-retaining insert, which was then upgraded to a cruciate-stabilising (CS) insert and upsized to a 14 mm insert. Acceptable mediolateral and anteroposterior stability was found after liner exchange. Postoperatively, there was neither further posterior sag nor knee recurvatum. Additional procedures (manipulation under anaesthesia) were performed in one knee for post-TKA stiffness. Eleven knees (3.7%) had minor wound complications (wound dehiscence/wound infection) requiring minor wound debridement or scar revision, but there were no PJIs recorded.

## Discussion

Our study demonstrated that early RLLs and bone resorption are not uncommon occurrences post-TKA. Most RLLs managed to resolve spontaneously and were preceded by trabecular line formation, pointing towards successful bone ingrowth and osteointegration. Bone resorption represented stress-shielding and occurred at a later postoperative course. Our study provides a simple and assessable method for monitoring osteointegration in cementless TKA, and could aid in preoperative counselling. The use of robotic-assisted cementless TKA is safe and effective with minimal postoperative complications and revisions in our cohort.

### Incidences of trabecular lines, radiolucent lines and bone resorption

Most included knees (90%) showed new bone ingrowth in the form of new trabeculae, which were signs of successful osteointegration. While previous studies have reported on areas of bone resorption as well as RLL formation, none have specifically investigated new bone formation regarding trabecular development. Mikashima et al. showed that up to 96% of knees had bone on-growth indicated by “white shadow” beneath the tibial tray but did not address trabecular formation [22]. Conversely, it is well established that trabecular lines are likely to be found at load-bearing regions in cementless total hip arthroplasty (THA), particularly around the distal stem region [23, 24]. Hence, we hypothesize that similar principles could apply to cementless TKA. In our patients, most trabeculae were seen medial and lateral to the tibial keel on AP view, and in the posterior area under the tibial tray on lateral view. These areas could represent areas of load-transfer, stimulating further new bone formation. Stoddart et al. showed that the regions medial and lateral to the tibial keel were often overstrained by up to a 100% difference compared to the native tibia [25]. Strain-loading increases bone mineral density while the opposite occurs in strain-shielded areas. These findings aligned with the principles of bone remodeling described by Wolff’s law, which suggested that bone is deposited and reinforced at areas of greatest stress [26]. Apart from axial compression, the tibial baseplate is subjected to anteroposterior and medial–lateral loading forces. While there has been a lack of studies documenting the relationship between trabecular bridging and biologic bone ingrowth in TKAs, it has been previously reported that the presence of bone apposition and trabecular bridging on serial imaging was associated with successful osteointegration in complex acetabular revision with bone loss [27]. Primary implant stability reduces micromotion during the early postoperative bone healing window. Excessive micromotion was shown to promote fibrous tissue interposition rather than biologic bone ingrowth [28]. Porous implants mainly promote surface-level bone ingrowth and allow for trabecular penetration into the metal scaffold, further consolidating biologic integration [29]. In obese patients with excessive stress on implants, excessive implant micromotion may occur, which hinders biologic fixation [30].

For RLLs, early RLLs are more commonly found in the femoral anterior chamfer region. Previous literature often reported RLLs in cemented TKA [12–15, 31–33] but lacked extensive evidence on cementless knees. It was reported that the incidence of RLLs in cemented TKAs ranged from 4.1% to 48% [12–15, 31–33]. Ng et al. demonstrated that up to 48% of cemented TKAs had physiological RLLs, commonly under the medial tibial baseplate (38%) [31]. RLLs in cemented and cementless TKA are not comparable due to intrinsic differences in their mechanism of formation [13]. RLLs in cemented TKA are likely due to failures at the bone-cement interface and might not manifest immediately postoperatively [34, 35]. Instead, they tend to form slowly and progress towards clinically significant loosening in severe cases. Cement particle residues might lead to the formation of an extra bone-cement interface (RLLs), but risk is mitigated in cementless TKAs [13]. As for cementless knees, recent literature demonstrated a varying RLL rate of 3.8% to 93% in the early postoperative period [13, 22]. We speculate that RLLs are most likely due to intraoperative factors and are more likely seen shortly after surgery. All TKAs in our study are robotic-assisted using the Mako-robotic arm system. Intraoperatively, a change of saw attachment piece may introduce inaccuracy in bone cuts and, consequently, increased rates of incomplete component seating [36, 37]. As a result, RLLs in cementless TKA may still be related to surgical technique and should be interpreted with caution. Most early RLLs were non-progressive and spontaneously resolved with new bone growth obliterating the bone-implant interface. Although our findings were limited to a single implant system, conclusions were supported by other studies with cementless TKA performed by alternative systems. Desmarais et al. reported 312 patients treated with the cementless Attune system (Depuy Synthes) and echoed that RLLs were not indicative of failed osteointegration and were uniformly < 1 mm and nonprogressive [12]. While the exact pathogenesis of RLLs in cementless TKA is unknown and our explanation is speculative, these could be attributed to implant design flaws and instrumentation errors leading to suboptimal bone-implant fixation [12, 38]. Non-progressive early RLLs are likely benign with spontaneous disappearance and do not contribute to increased revision rates [22, 31].

In addition, approximately half of the included knees exhibited variable degrees of resorption. Bone resorption may be attributed to stress shielding, following principles similar to those observed in cementless THA [39, 40]. Biomechanical studies have shown that most significant stress shielding occurs under the medial tibial tray and distal femur, aligning with the common areas of bone resorption in our study [25]. In our knees, new bone formation occurred medial and lateral to the tibial keel. The increase in load bearing in these regions translated to proximal stress shielding in the form of resorption often under the medial or lateral tibial tray. Previous studies also echoed that resorption commonly occurred under the medial tibial baseplate [41–43]. However, almost none of the knees with bone resorption had clinically significant aseptic loosening warranting revision surgery. Hence, the presence of bone resorption is possibly a benign sign of load transfer early in the postoperative course. Given that the longest follow-up was at > 5-years, whether bone resorption translates to detrimental complications requires further long-term investigation.

### Temporal pattern of osteointegration

Process of biological fixation in cementless TKAs with early RLLs became apparent by analysing the sequence of osteointegration events. Typically, new bone formation initiates early postoperatively, often within the first 3-months. When bone ingrowth is sufficient to obliterate the bone-implant gap, the disappearance of RLLs follows. This new bone ingrowth plays a pivotal role in effectively securing the implant. In areas of more secure fixation, increased stress-loading may contribute to the stress shielding effect in neighboring zones. In the long-term, stress-shielding leads to zones of bone resorption [41–44]. Knees with early bone in-growth were more likely to witness RLL resolution in the earlier postoperative period. Most RLLs disappeared within the first 12-months, accompanied by the formation of most trabecular lines (Fig. 4A and B), indicating that this timeframe is for achieving solid fixation after cementless TKA. Interestingly, earlier trabecular line formation was observed in knees without immediate postoperative RLLs. Our initial speculation on early RLL formation revolved around intraoperative inaccurate bone cuts and incomplete component seating. We hypothesize that more accurate component seating in these patients could facilitate trabecular formation with increased implant-bone contact.

Understanding the timeframe and temporal pattern of osteointegration can provide valuable guidance to surgeons, highlighting specific radiographic features to monitor during subsequent follow-ups. For example, the patient should be more closely monitored for any signs of loosening if new trabecular lines are absent or early RLLs failed to resolve within the initial 12-months postoperatively. While RSA remains the gold standard for assessing osteointegration and implant migration, its use is limited by the need for intraoperative marker bead insertion and specialized radiographic equipment [10]. Although using plain radiographs for assessing osteointegration may be subject to interobserver differences in the hands of different interpreters and might not be as precise as RSA, we believe that this approach provides a simpler and more assessable way for postoperative surveillance in the wider TKA population. Surgeons should know the limitations of this approach; suspicions for implant loosening/poor bone ingrowth at postoperative follow-ups should be adequately followed up with more advanced investigations like CT scans.

### Relationships of factors with osteointegration

Failure to demonstrate significant associations between preoperative factors and osteointegration could be explained by baseline characteristics of our cohort. Only 2.3% of patients were osteoporotic, and only 3 patients were receiving bisphosphonate treatment. Low prevalence of osteoporotic patients and those receiving antiresorptives could potentially limit the reliability of relationship analysis relating to RLL disappearance, bone resorption, and trabecular line formation. These could be due to underdiagnosis of osteoporosis in TKA patients, as routine DEXA for osteoporosis screening was not performed in our institution. Osteoporosis has been well-recognized as a risk factor for suboptimal bone quality and subsequent revision. Recent studies have proposed that osteoporosis may not be contraindicated, as both cementless and cemented TKA showed similar mid-term clinical outcomes [45]. However, if preoperative bone quality is severely compromised, initial cemented fixation may be more reliable for achieving primary stability, as greater implant micromotion was expected in osteoporotic knees, hindering osteointegration [46]. The long-term use of bisphosphonates also significantly reduces revision risk by reducing periprosthetic bone loss and implant migration [16, 47]. Increased age is associated with poorer fixation and higher risk of aseptic revision [47]. However, our regression analysis paradoxically showed that increased age was a positive predictor for trabecular line formation. It was well known that increased age was related to reduced bone metabolism, increased bone loss, and lower bone mineral density [48]. Given that routine DEXA scans were not performed in our cohort and the potential underdiagnosis of osteoporosis, it was not possible to assess for individual bone quality in relation to age. Hence, this seemingly paradoxical relationship might simply be a chance statistical association driven by unmeasured confounding, including baseline bone mineral density. Alternatively, trabecular line formation may appear more conspicuous on radiographs in older patients with reduced BMD, potentially due to increased baseline radiolucency in the peri-implant regions. Future studies should examine the relationship between age and osteointegration while adjusting for relevant comorbidities and osteoporosis status. The literature also had inconsistent reports on the effect of smoking and alcohol consumption on osteointegration. While smoking is expected to negatively affect osteointegration, studies have reported a lower risk of aseptic loosening in drinkers [49]. Only around 10% of patients were either smokers or drinkers, and both were not shown to associate with osteointegration in our study. Although hypertension was initially shown to be protective for bone resorption in univariate analysis, the relationship was not significant after multivariate adjustment. Osteoblast differentiation is stimulated in patients taking angiotensin receptor blockers or selective adrenergic receptor blockers for hypertension control, thereby increasing trabecular bridging and bone mineral density [50], emerging as a hidden confounder in the current relationship analysis.

### Mid-term clinical outcomes

Only two knees in our entire cohort had component loosening, and only two knees required revision. Previously, there were concerns about whether the absence of initial cement fixation could lead to increased component instability [51]; our results showed that this is not the case, which echoed recent studies in favor of uncemented fixation [52, 53]. Cementless knees demonstrated very low rates of aseptic loosening with effective biological fixation, simultaneously mitigating the risks of cement-related complications [6]. No RLLs are associated with clinically significant loosening, and there were no cases of PJIs. Hence, robotic-assisted cementless TKAs could safely act as an effective alternative to traditional cemented fixation.

### Limitations

Although the precision of using plain radiographs for monitoring osteointegration is limited by the presence of more advanced technologies such as RSA and artifact-reduction CT, notable strengths of our study included a large patient cohort of the same implant system. Serial monitoring of radiographs to determine the temporal relationships of RLLs, trabecular ingrowth, and bone resorption was a novel contribution to the growing interest in cementless TKAs. However, our study does have limitations. First, the nature of our study involved a single institution, single robotic platform, and a single implant system. Findings on the temporal relationship and incidences of RLLs/trabecular lines were limited to the highly porous titanium-coated implant utilized, and further studies involving comparisons with other implant systems are warranted to verify the generalizability of our findings to other TKA platforms. All investigators are also from the same institution. Despite being blinded to patient information during subsequent radiographic measurements, potential institutional bias may result. The presence of external investigators in the form of a multi-centre study may strengthen study conclusions. Second, patient-reported outcome measures (PROMs) were not documented. The nature of our study was mainly radiographic. The inclusion of PROMs in future prospective work is important to establish the clinical relevance of observed radiographic changes. Further, the lack of variability in patient baseline characteristics limited relationship analysis. Apart from analytical constraints, the lack of routine preoperative screening for osteoporosis might act as a fundamental limitation in the reliability of relationship analysis, as there was underdiagnosis of osteoporotic patients in the study cohort. There was also potential confounding between the osteoporotic cohort and those with antiresorptive drug use. This was particularly important as osteoporosis has been shown to affect biologic fixation and implant micromotion. Implant sizing and mechanical alignment are also critical determining factors for bone resorption. For instance, larger tibial trays cause more proximal stress shielding [54]. Future studies incorporating these biomechanical and intraoperative factors would make the relationship analysis for osteointegration more comprehensive. All patients underwent a standardized rehabilitation regimen, and additional studies correlating different rehabilitation strategies/timing of weight-bearing with osteointegration may be warranted to determine how these factors influence progression of osteointegration. Furthermore, future studies incorporating both radiographs and more advanced measurements (e.g., RSA) are highly recommended to verify the reliability of the radiographic features signifying osteointegration. Last, analyses were done treating knees as independent observations and did not adjust for within-patient correlations, potentially affecting variance estimation.

## Conclusion

In highly porous cementless TKAs with a 3D-printed titanium-coated implant system, bone ingrowth in the form of new trabeculae was observed in > 90% of knees, pointing towards satisfactory osteointegration. Osteointegration typically occurred between 4.5 and 8.9 months with trabecular line formation and RLL disappearance. Bone remodeling from the stress shielding effect continued beyond 14 months, leading to zones of bone resorption. Our study provided a simple and assessable alternative to monitoring biologic fixation after cementless TKA, and this temporal relationship can aid surgeons’ preoperative counselling.

## Data Availability

The datasets used and/or analyzed during the current study are not publicly available but are available from the corresponding author on reasonable request.

## Abbreviations

TKA: Total knee arthroplasty
RLL: Radiolucent line
OA: Osteoarthritis
RSA: Radiostereometric analysis
rKA: Restricted kinematic alignment
MCL: Medial collateral ligament
AP: Anteroposterior
MPTA: Medial proximal tibial angle
LDFA: Lateral distal femoral angle
PJI: Prosthetic joint infection
OR: Odds ratio
B: Beta coefficient
PROMS: Patient-reported outcome measures
BMI: Body-mass index
CI: Confidence interval
SD: Standard deviation
THA: Total hip arthroplasty
DEXA: Dual-energy X-ray absorptiometry
ICC: Intraclass correlation coefficient
